# Acrylamide Neurotoxicity as a Possible Factor Responsible for Inflammation in the Cholinergic Nervous System

**DOI:** 10.3390/ijms23042030

**Published:** 2022-02-12

**Authors:** Marta Kopańska, Anna Łagowska, Barbara Kuduk, Agnieszka Banaś-Ząbczyk

**Affiliations:** 1 Department of Pathophysiology, Institute of Medical Sciences, Medical College of Rzeszow University, 35-959 Rzeszow, Poland; 2 Students Science Club “Reh-Tech”, University of Rzeszow, 35-959 Rzeszow, Poland; alagowska7@gmail.com (A.Ł.); barbarakuduk@wp.pl (B.K.); 3 Departament of Biology, Institute of Medical Sciences, Medical College of Rzeszow University, 35-959 Rzeszow, Poland; agnieszkabanas@o2.pl

**Keywords:** acrylamide, cholinergic nervous system, inflammatory response

## Abstract

Acrylamide (ACR) is a chemical compound that exhibits neurotoxic and genotoxic effects. It causes neurological symptoms such as tremors, general weakness, numbness, tingling in the limbs or ataxia. Numerous scientific studies show the effect of ACR on nerve endings and its close connection with the cholinergic system. The cholinergic system is part of the autonomic nervous system that regulates higher cortical functions related to memory, learning, concentration and attention. Within the cholinergic system, there are cholinergic neurons, anatomical cholinergic structures, the neurotransmitter acetylcholine (ACh) and cholinergic receptors. Some scientific reports suggest a negative effect of ACR on the cholinergic system and inflammatory reactions within the body. The aim of the study was to review the current state of knowledge on the influence of acrylamide on the cholinergic system and to evaluate its possible effect on inflammatory processes. The cholinergic anti-inflammatory pathway (CAP) is a neuroimmunomodulatory pathway that is located in the blood and mucous membranes. The role of CAP is to stop the inflammatory response in the appropriate moment. It prevents the synthesis and the release of pro-inflammatory cytokines and ultimately regulates the local and systemic immune response. The cellular molecular mechanism for inhibiting cytokine synthesis is attributed to acetylcholine (ACh), the major vagal neurotransmitter, and the α7 nicotinic receptor (α7nAChR) subunit is a key receptor for the cholinergic anti-inflammatory pathway. The combination of ACh with α7nAChR results in inhibition of the synthesis and release of pro-inflammatory cytokines. The blood AChE is able to terminate the stimulation of the cholinergic anti-inflammatory pathway due to splitting ACh. Accordingly, cytokine production is essential for pathogen protection and tissue repair, but over-release of cytokines can lead to systemic inflammation, organ failure, and death. Inflammatory responses are precisely regulated to effectively protect against harmful stimuli. The central nervous system dynamically interacts with the immune system, modulating inflammation through the humoral and nervous pathways. The stress-induced rise in acetylcholine (ACh) level acts to ease the inflammatory response and restore homeostasis. This signaling process ends when ACh is hydrolyzed by acetylcholinesterase (AChE). There are many scientific reports indicating the harmful effects of ACR on AChE. Most of them indicate that ACR reduces the concentration and activity of AChE. Due to the neurotoxic effect of acrylamide, which is related to the disturbance of the secretion of neurotransmitters, and its influence on the disturbance of acetylcholinesterase activity, it can be concluded that it disturbs the normal inflammatory response.

## 1. Introduction

The nervous system and the immune system interact strongly with each other within the human body. This interaction has a significant impact on maintaining the body’s homeostasis. These systems protect the body against pathogens and negative environmental factors. Numerous scientific studies show that the immune system influences the activity of the central nervous system in the same way as information from the brain controls the body’s immune function. Balance is most important in the proper functioning of the immune system, as both too little and too much activity can lead to pathology. This is where the nervous system plays a very important role, controlling and modulating the activity of the immune system. The mechanism of the inflammatory reflex is one example of the immunosuppressive action of the nervous system. The cooperation of these two systems is based on the production of special mediators such as cytokines, neuropeptides, and neurotransmitters. Lymphocytes and macrophages produce cytokines, but they can also secrete special mediators such as neuropeptides and opioids. The cells of the nervous system produce neuropeptides and neurotransmitters, and the expression of cytokines in the brain is also observed. In addition, both systems have cell receptors for transmitters, which allows them to communicate with each other. The autonomic nervous system innervates the peripheral and central lymphatic organs. Nerve endings release neurotransmitters that bind to receptors in the immune system, regulating its function. Many substances of external origin can disturb the balance between the two systems. This harmful effect is attributed, inter alia, to acrylamide.

## 2. Aim

The aim of the study was to review the current state of knowledge of the influence of acrylamide on the cholinergic system and its possible effect on inflammatory processes.

## 3. Acrylamide

Acrylamide (ACR) is an organic chemical compound with the chemical formula C3H5NO. It is composed of carbon (50.69%), hydrogen (7.09%), nitrogen (19.71%) and oxygen (22.51%) atoms. At room temperature, it is an odorless, crystalline solid with a molecular weight of 71.08, a melting point of 84.5 °C and a density of 1.122 g/cm^3^ at 30 °C. Due to its relatively low volatility, its boiling point is 192.6 °C at a pressure of 1 atm (101.3 kPa). Due to the presence of functional groups, this compound is polar and very soluble both in water and in other polar solvents such as methanol or ethanol. However, it is insoluble in benzene and heptane [1]. Acrylamide is a very reactive organic compound with a conjugated double bond and an amide fragment in its structure. Its high chemical activity is mainly due to the presence of a multiple bond that has electrophilic properties. The electrophilic center, whose role is played by a double bond, is susceptible to nucleophilic attack of the amino (-NH2) or sulfhydryl (-SH) groups of amino acids, peptides and proteins. Acrylamide can also form hydrogen bonds due to the presence of an amide group [2]. Due to the presence of both the vinyl and amide groups, it undergoes nucleophilic addition reactions, Diels-Alder reactions or Michael reactions. It has both acidic and slightly alkaline properties. Thanks to the amide group, it also undergoes hydrolysis, dehydration, alcoholization and condensation reactions with other aldehydes. It polymerizes when exposed to UV rays. It is stable at room temperature, but to meet this condition, it must be stored in a dark and cool place. The monomeric form of ACR is a white powder often used in chemical and industrial processes [3]. Currently, acrylamide is known not only as a synthetic material used in industry, but also as a toxic compound that is formed during the thermal processing of food (Maillard reaction) containing, among other substances, starch and carbohydrates—for example: potatoes, bread, plant products or coffee (Table 1) [4]. It has been shown that acrylamide is formed in food during its processing at a temperature above 100 °C from nitrogen-containing compounds or oils. The presence of acrylamide in so many of the most commonly consumed foods makes human exposure to this toxin inevitable. In 1994, the International Agency for Research on Cancer recognized acrylamide as a probable human carcinogen [5]. ACR is also neurotoxic and genotoxic. During studies on animals that received acrylamide, neurological symptoms in the form of tremors, general weakness, numbness, tingling in the limbs or ataxia were observed [6]. The results of numerous experiments indicate that nerve endings are the site of acrylamide action. Early and progressive degeneration of nerve endings has been noted in all areas of the CNS. The long-term effect of acrylamide may cause irreversible changes in the nervous system and disturbance of the transmission of impulses between neurons [7]. In addition, studies show that acrylamide in low doses causes oxidative stress in various organs [8]. 

Currently, a maximal limit for the ACR’s contents in food products has not yet been specified. In accordance with the United Union’s provisions, potable water’s maximum allowable concentration of ACR is 0.1 µg/dm^3^. However, the Environmental Protection Agency (EPA) guidelines set the amount at 0.5 µg/dm^3^. The maximal ACR’s residuum in the case of cosmetics shall not exceed 100 µg/kg [3], whereas the allowable ACR’s concentration in a production plant’s air shall not exceed 30 µg/m^3^ [1].

## 4. The Cholinergic System

**Figure 1:**
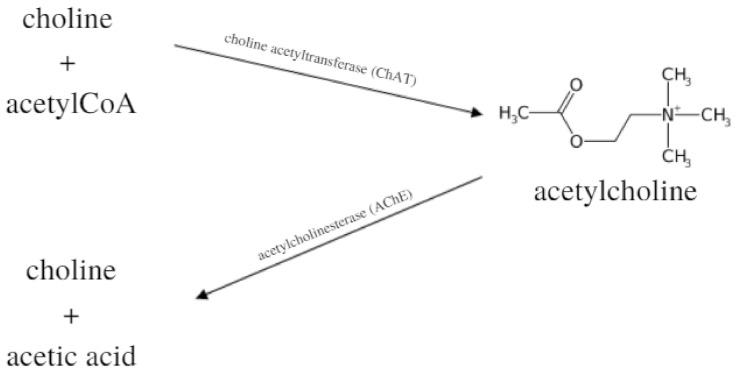
The synthesis and metabolism of the acetylcholine.

Acetylcholine is a ubiquitous neurotransmitter [9,10]. It occurs even in the organism with the simplest nervous system, the roundworm (*Caenorhabditis elegans*) [11]. In roundworms, the cholinergic system constitutes one-third of the entire nervous system [12]. A large percentage of the nervous system in humans is cholinergic, including the CNS. The cholinergic nerves also make up a large part of the parasympathetic and sympathetic nervous systems [13]. They include cholinergic neurons, anatomical cholinergic structures, the neurotransmitter acetylcholine ACh and cholinergic receptors (Figure 1). Acetylcholine is synthesized from choline and acetyl coenzyme A, and the catalyst for this synthesis taking place in nerve endings is Choline Acetyltransferase (ChAT) [14]. The resulting acetylcholine is stored in synaptic vesicles, and then, as a result of depolarization of the presynaptic membrane, ACh is released into the synaptic cleft. From there, it acts on the muscarinic and nicotinic receptors present on the postsynaptic membrane. After detaching from the ACh receptor, it is broken down into choline and acetic acid by acetylcholinesterase-AChE. The resulting choline is reabsorbed into the presynaptic terminals of the neurons. Inhibition of AChE increases the concentration of ACh in the synapse and triggers a response from muscarinic and nicotinic receptors [15]. Many non-neuronal cells in the body are capable of synthesizing ACh. This is due to the fact that the presence of ChAT has been demonstrated, for example, in macrophages or lymphocytes [16,17]. Both the cells of the nervous and immune systems are able to synthesize various mediators (e.g., neurotransmitters, cytokines) and additionally have receptors for these mediators on their cell membranes. The immune system response is coordinated and modulated by the autonomic nervous system, which innervates the central and peripheral lymphatic organs. Acetylcholine binds to nicotinic receptors on the macrophage cell membrane and induces a signal transduction pathway that reduces the synthesis of pro-inflammatory cytokines by these cells and inhibits inflammation [18,19]. Some scientific reports suggest that acrylamide may disrupt the synaptic conduction of cholinergic neurons in the central and peripheral nervous system by influencing the activity of acetylcholinesterase. These changes may be related to the pro-oxidative properties of acrylamide [20].

## 5. Cholinergic Anti-Inflammatory Pathway

The cholinergic anti-inflammatory pathway (CAP) is a neuroimmunomodulatory pathway located predominantly in the blood and mucosa in which acetylcholine (ACh) is released through the interaction of the vagus nerve with the α7 nicotinic acetylcholine receptor (α7nAChR). It prevents the synthesis and release of pro-inflammatory cytokines or ultimately regulates systemic inflammatory response by feedback. A proper immune response against harmful antigens is essential to maintaining immune homeostasis. However, an exaggerated immune response usually causes the tissue damage that accompanies autoimmune or immunopathological disorders. For this reason, regulatory mechanisms that modify innate and adaptive immunity are needed to limit this damage. For a long time, mechanisms of regulating inflammation remained unclear. The discovery of the anti-inflammatory cholinergic pathway has made it possible to understand how the CNS is involved in the regulation of innate immunity [21]. The cellular molecular mechanism for inhibiting cytokine synthesis is attributed to acetylcholine (ACh), the major neurotransmitter of the vagus nerve [22,23,24]. Studies have shown that the α7nAChR is a key receptor for the cholinergic anti-inflammatory pathway, expressed mainly by macrophages, but also by lymphocytes and microglial cells [25,26,27]. The combination of acetylcholine with this receptor subunit causes inhibition of the synthesis and release of pro-inflammatory cytokines [28,29]. The mechanism of the above-mentioned anti-inflammatory effect is based on the inhibition of the activity of the nuclear transcription factor NF-κB, which is the most important for the formation of pro-inflammatory cytokines. Moreover, acetylcholine, by binding to the α7nAChR receptor, stimulates the anti-inflammatory cytokine synthesis pathway (JAK-STAT) by activating the transcription factor STAT3. Activation of the vagus nerve stimulates the secretion of acetylcholine, which inhibits the production of pro-inflammatory cytokines, including tumor necrosis factor (TNF-alpha), IL-1Beta and IL-6 by lipopolysaccharide-activated macrophages (LPS) [25,28,29], but it does not inhibit the production of IL-10, the anti-inflammatory cytokine [26]. In a rat model of endotoxemia, electrical stimulation of the vagus nerve has been shown to significantly attenuate the release of pro-inflammatory cytokines and consequently prevent the development of shock, which develops the concept of the anti-inflammatory cholinergic pathway as a neuroimmunological interaction [17]. In the above process, Huston et al. showed that this suppressive effect is canceled by spleen resection, indicating the need for the spleen to be present in the cholinergic anti-inflammatory pathway during systemic inflammation [30]. The vagus nerve inhibits cytokine activities and alleviates the disease, especially in an end-stage disease in experimental models of sepsis, ischemia/reperfusion, hemorrhagic shock, myocardial infarction, intestinal obstruction, arthritis, and pancreatitis [17,31,32,33,34]. 

## 6. Description of Acetylcholinesterase and the Inflammatory Response

**Figure 2:**
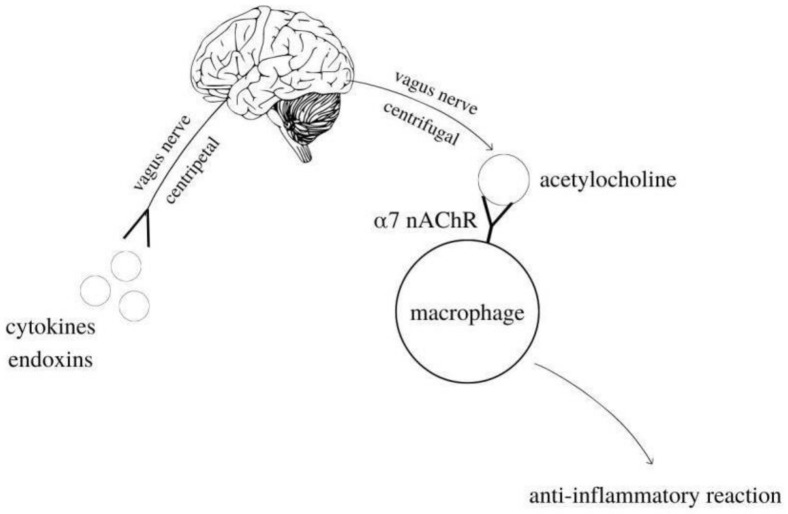
Inflammatory reflex. Cholinergic anti-inflammatory pathway.

Acetylcholinesterase can terminate the stimulation of the cholinergic anti-inflammatory pathway due to the cleavage of ACh [35]. One study investigating the effect of AChE activity on inflammatory response investigated changes in brain cholinergic gene expression and related immune responses during polycarpine-induced epilepsy in mice. As early as 48 hours after polycarpine administration, AChE expression was observed in hippocampal neurons, microglia, and endothelial cells. Mice with increased AChE expression showed increased levels of pro-inflammatory cytokines. This study showed that ACh directly inhibits the innate immune response of the brain and that elevated AChE levels are associated with increased immune response [36]. Other studies have found that ACh is a neurotransmitter that regulates the levels and activity of serotonin, dopamine, and other neuropeptides, thereby modulating the immune response and neurotransmission. Therefore, AChE may increase inflammation by inactivating ACh. These findings suggest that increased plasma and tissue AChE activity in various clinical conditions may serve as a low-grade systemic marker of inflammation [37]. Studies on the expression and function of the cholinergic system in immune cells have shown that the nicotinic receptors (α7nAChR) are involved in the regulation of cytokine production and thus modulate antibody production. It is one step in the cholinergic anti-inflammatory pathway (Figure 2). 

The authors of the study also state that AChE is expressed ubiquitously in murine lymphocytes, DCs, and macrophages. In addition, elevated AChE activity is detected in peripheral blood lymphocytes. The study also indicated that AChE inhibition increases the level of ACh, which acts on the α7nAChR [38]. Similarly, in another study, cholinergic enzymes and markers of inflammation were assessed. Its aim was to assess the activity of AChE in serum, whole blood, and lymphocytes and to verify its relationship with the immune response in experimentally infected rats. Rats were divided into three groups: negative control, infected subcutaneously, and infected intraperitoneally. On certain days after infection, the activity of AChE in lymphocytes and whole blood, the level of cytokines, the level of immunoglobulins and the protein profile by electrophoresis were assessed. The infected groups showed elevated levels of inflammatory parameters. AChE activity in lymphocytes increased significantly, especially in the intraperitoneally infected group. Therefore, it was found that AChE plays an important modulating role in the immune response [39]. In turn, another study aimed to test the influence of the cholinergic and adenosine systems on the immune response and inflammatory process of experimentally infected fish. As a result of the infection, a decrease in AChE activity was observed, while the level of ACh was increased. It was found that reducing AChE activity exerts an anti-inflammatory profile [40]. Based on the above, it can be concluded that cytokine production is essential for protection against pathogens and promoting tissue repair, but the over-release of cytokines can lead to systemic inflammation, organ failure, and death. Inflammatory responses are precisely regulated to effectively protect against harmful stimuli. The central nervous system dynamically interacts with the immune system, modulating inflammation through the humoral and nervous pathways (Table 2). Autonomous regulation of local and systemic inflammation is mediated by the “cholinergic anti-inflammatory pathway”, a mechanism consisting of the vagus nerve and its major neurotransmitter acetylcholine, a process dependent on the α7nAChR [25]. The stress-induced rise in acetylcholine (ACh) acts to ameliorate the inflammatory response and to restore homeostasis. This signaling process ends when ACh is hydrolyzed by acetylcholinesterase (AChE) [41]. 

## 7. Effect of Acrylamide on Acetylcholinesterase

Acrylamide is a neurotoxin whose degenerative effect on the cholinergic system has been demonstrated in numerous scientific studies. They mainly concern the influence of acrylamide on the activity of acetylcholinesterase. One such study looked at the effect of acrylamide on acetylcholinesterase activity in the hypothalamus, myocardium, thigh skeletal muscle, and small intestine smooth muscle. Acrylamide was injected intraperitoneally into mice. The activity of acetylcholinesterase was studied based on the concentration of thiol groups and malondialdehyde in these structures. The results showed a significant decrease in AChE activity in muscles and the hypothalamus, which indicates a direct effect of acrylamide on peripheral nerves, causing structural damage and physiological changes [20]. Other studies investigated changes in axonal transport and the content of different molecular forms of AChE in nerve and muscle tissues of healthy chickens as a result of acrylamide treatment. In ACR poisoned sciatic nerves, a marked reduction in transport of two of the major forms of AChE was observed. These forms were considered to be very sensitive markers of both the phases of axon transport and the innervation state of the studied structures [42].

There have also been studies investigating the effect of different doses of acrylamide on some enzymatic activities in male rats. The animals were randomized into groups administered with various concentrations of acrylamide for 10 weeks. It was observed that acrylamide significantly decreased the activity of creatine kinase, the level of plasma proteins as well as the activity of acetylcholinesterase in the plasma and the brain. These results indicate that different doses of acrylamide exerted the effect of deteriorating enzyme activity in a dose-dependent manner [8]. Similar conclusions were drawn from studies designed to evaluate the toxic effects of ACR on the activity of the gastrocnemius motor plate in rats. In these animals, randomly divided into groups, changes in the structure of muscle fibers and nerve endings were observed. The muscle fibers became thinner and the muscle fibers became short and bright; the number of neuromuscular plates decreased. A lower AChE content was also observed [43]. Similarly, the effect of acrylamide on acetylcholinesterase has been noted in studies with electric eel. It concerned the capture of acetylcholinesterase from electric eel in a polyacrylamide gel. The activity of the trapped enzyme was significantly reduced; the effect was due to acrylamide inhibition [44].

The authors of another study (Table 3) carried out on adult male mice concluded that ACR induced oxidative stress and related neurotoxic effects. Dose-dependent exposure of the ACR tested animals resulted in a higher death rate, significant locomotor deficits and oxidative stress, especially in the head regions. In contrast to the results of other research, in this study, however, increased activity of acetylcholinesterase and depletion of dopamine concentration were observed. Therefore, it can be concluded that there is a need for more studies on the effects of ACR on acetylcholinesterase in order to be able to establish the actual impact of ACR on the activity of this enzyme [45].

**Figure 3:**
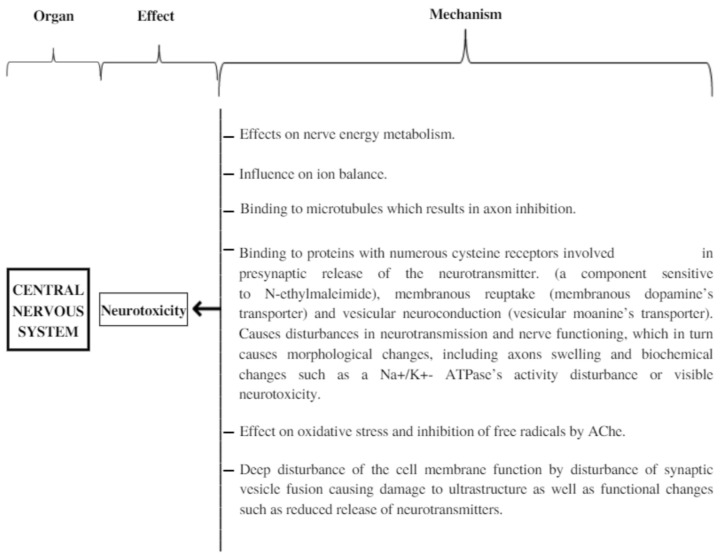
Mechanisms leading to neurotoxicity.

It would seem essential to understand the neurotoxicity’s mechanism, for such a system has been observed not only in animals but also in the ACR’s exposed population (Figure 3). There was an accident of ACR and N-methylacrylamide’s leakage in 1997 in Sweden, which happened during the construction of a tunnel. Acrylamide neurotoxic influence on humans was confirmed at that time [20]. Neurological observations were made of distal axon edema and degeneration of axons in the central and peripheral nervous system. This was a distinctive neuropathological characteristic change caused by ACR. This has resulted in the qualification of an ACR-caused neuropathy as a central–peripheral axonopathy. Nowadays, it is thought that nerve endings, and not axons, are the primary location of the ACR’s activity. The research results, which were obtained out of animal experiments, could ascertain the early and progressive degeneration of nerve endings in all of the central nervous system (CNS) areas as well as damage to the cerebellum’s Purkinje cells. Furthermore, it has been found that axon degeneration was a secondary process [20]. The brain and the great sciatic nerve’s creatine phosphokinase (CPK) are characterized by a particular ACR sensitivity that blocks its activity. The result of this enzyme’s inhibition is adenosine-5’-triphosphate (ATP) deficiency in a cell, which as a consequence can result in apoptosis. It is worth noting that some experimental studies proved the CPK activity in a human’s brain is entirely inhibited by ACR. It is proof that the human brain is extremely sensitive to ACR. The prolonged contact with ACR may result in irreversible CNS damage through neuronal impulse inhibition [20]. The toxicity of ACR is related to the duration of exposure. It also depends on the injection method and the length of time in the body. We can conclude that the longer the exposure time, the greater the damage.

## 8. Effect of ACR on the Inflammatory Response through Its Action on Acetylcholinesterase

Many scientific studies show that exposure to ACR can trigger an inflammatory response in the body. It is based on the relationship between ACR and the cholinergic nervous system. Zhao et al. confirmed the hypothesis that ACR induces Nrf2 and NF-κB pathways related to oxidative stress and cytokine release in primary astrocytes and microglia. These results explain the role of these pathways in ACR-induced neurotoxicity [47]. Another study also indicated the neurotoxic effects of acrylamide by inducing oxidative stress and apoptotic and inflammatory responses in the microglia in mice. Moreover, it has been shown that ACR disturbs the energy metabolism of cells by reducing mitochondrial respiration and apoptosis in BV2 microglial cells. Mitochondrial dysfunction mediated by ACR and a more oxidized redox state lead to an intrinsic apoptotic pathway and inflammatory responses [48]. Other scientific studies show that the exposure to ACR induces inflammatory responses in the cerebral cortex through increased expression of mRNA and increased pro-inflammatory cytokine proteins. In addition, ACR induced microglia activation, as indicated by the increased expression of its markers. Studies with BV2 microglial cells confirmed the inflammatory response of the microglia [49]. Ekuban et al. conducted their study to determine the role of Nrf2 in ACR-induced neurotoxicity. The results show the susceptibility of Nrf2 knockout mice to acrylamide-induced neurotoxicity and microglial activation neurotoxicity. Moreover, the role of Nrf2 has been suggested not only in the induction of antioxidant gene expression, but also in the suppression of the expression of pro-inflammatory cytokine genes. The authors of the study indicate the quite specific impact of ACR, indicating that it induces the degeneration of monoaminergic neurons, including noradrenergic and serotonergic neurons. However, there is too little scientific research to be able to clearly demonstrate the specific effects of ACR; therefore, there is a need to continue research into the specificity of ACR-induced damage in the body [50]. Moreover, acrylamide disrupts redox metabolism by reducing the amount/activity of antioxidants such as reduced glutathione, catalase and superoxidismutase [46]. Imbalance between production of free radicals and antioxidants leads to damage of important biomolecules and cells. Oxidative stress can activate a variety of transcription factors and lead to the expression of over 500 different genes, including those for growth factors, inflammatory cytokines, chemokines, cell cycle regulatory molecules, and anti-inflammatory molecules [51]. Furthermore, mRNA expression levels of NFĸB, IFN-γ, IL-1β, and TNF-α in the liver and brain of rats were increased under ACR treatment [52]. Also, acrylamide induces NLRP3 inflammasome activation via oxidative stress- and the endoplasmic reticulum stress-mediated MAPK pathway in HepG2 cells [53].

It’s important to take a look at the research of Sui Xin et al., who performed experiments on C57BL/6 mice. Acrylamide exposure caused significant activation of NLRP3 inflammasomes and neuroinflammation, whereas inhibiting these effects through specific NLRP3 inflammasome blocker MCC950 intervention or NLRP3 knockout significantly ameliorated AA-induced ataxia, cerebellar Purkinje cell degeneration and apoptosis. Their findings indicate that activation of the NLRP3 inflammasome pathway is involved in AA-induced neurotoxicity, whereas MCC950 treatment or NLRP3 knockout could effectively protect against AA-induced neurotoxic injury through the inhibition of neuroinflammation and activation of the Nrf2 antioxidant pathway [54]. Due to the neurotoxic effect of acrylamide, which is related to the disturbance of the secretion of neurotransmitters, and the previously shown influence on the disturbance of acetylcholinesterase activity, it can also be assumed that it interferes with the normal inflammatory response. The appropriate concentration and action of AChE are closely related to the regulation of cholinergic conduction and directly proportional to the amount of ACh acetylcholine secreted, which, in turn, is essential for the functioning of the cholinergic anti-inflammatory pathway. It follows that if acrylamide inhibits and reduces the concentration of acetylcholinesterase, it will thereby cause a proportional increase in acetylcholine concentration and potentiation of acetylcholine neurotransmission. This, however, if it potentiates the cholinergic anti-inflammatory pathway, can be detrimental for a correct inflammatory response, as the prolonged activation leads to a7 nAChR desensitization and therefore to impairment of the ability to respond to subsequent inflammatory insults, resulting in excessive and chronic inflammation. Acrylamide causes an inflammatory response with neuronal damage as a consequence. However, we cannot exclude that it is nerve damage first and consequently an inflammatory response. There are still few reports that would definitely demonstrate this mechanism; therefore, there is a need for further research in this area.

## 9. Conclusions

Studies on the harmful effects of acrylamide and its metabolites indicate three possible types of toxicity: neurotoxicity, genotoxicity, and carcinogenicity. So far, only the neurotoxic effect of acrylamide on the human body has been undoubtedly proven. The studies conducted so far indicate, first of all, the reduction of acetylcholinesterase activity. The genotoxic activity of acrylamide is mainly manifested after its metabolic conversion to the epoxide derivative glycidamide. The carcinogenic effect of acrylamide has been demonstrated in animal studies. For humans, the exposure to acrylamide is assessed by measuring adducts, i.e., specific compounds formed by combining acrylamide with hemoglobin or DNA. There is a need for further epidemiological studies that will provide irrefutable evidence that acrylamide ingested with the diet can initiate the formation of cancer in humans and induce an inflammatory response.

## Figures and Tables

**Table 1:** The content of acrylamide in food products.

The Products Type	The Acrylamide Content (µg/kg)
Bread (rolls, bread, bagels)	70–430
Potato chips	<50–3500
Potato fries	200–2287
Boiled potatoes	48
Cookies, crackers, biscuits	<30–3200
Rusks	80–1200
Cereals	30–1400
Gingerbread cookies	<50–100
Chocolate (powder)	64–457
Nuts, peanut butter	64–457
Meat, poultry	30–64
Baked asparagus	143

**Table 2:** Acetylcholinesterase and inflammatory response.

Authors	Aim	Methods	Results	Conclusions
Gnatek et al. [36]	Changes in the brain cholinergic gene expression and related immune responses during pilocarpine-induced epilepsy.	AChE levels and inflammatory markers were measured during pilocarpine-induced seizures in mice.	the action of pilocarpine leads to an increase in the level of AChE	Increased AChE levels are associated with an increased immune response. The cholinergic system is a potential area that can be influenced in order to reduce epileptic seizures.
Castro et al. [39]	AChE activity in serum, blood and lymphocytes and its relationship to the immune response after induced inflammation	The rats were divided into three groups that were infected by different routes. AChE activity in blood and lymphocytes was assessed at 15, 30 and 40 days after infection.	A significant increase in AChE activity was observed, especially in the intraperitoneally infected group.	AChE plays an important role in modulating the immune response.
Baldissera et al. [40]	Influence of the cholinergic system on the immune response and inflammation in the gills.	Ach levels in the gills of silver catfish were compared in fish with/without induced inflammation	Reduced acetylcholinesterase activity and increased acetylcholine levels in the gills of infected animals were observed.	the inflammatory process alters the cholinergic system, suggesting a contribution of AChE activity to the immune and inflammatory response by regulating ACh levels. The reduction in AChE activity exerts an anti-inflammatory profile.

**Table 3:** Effect of Acrylamide on Acetylcholinesterase.

Authors	Aim	Description of the Study	Results	Conclusions
Kopańska et al. [46]	Assessment of the effect of Acrylamide on the activity of Acetylcholinesterase in the hypothalamus, myocardium, skeletal muscles of the thigh and smooth muscles of the small intestine.	The effect of Acrylamide on the activity of Acetylcholinesterase in the hypothalamus, myocardium, skeletal muscles of the thigh and smooth muscles of the small intestine was measured depending on the thiol groups and the concentration of malondialdehyde. Swiss mice were used for the study. ACR was injected intraperitoneally at various doses.	AChE activity was significantly lower in all structures studied. The greatest decrease of 75.09% was observed in the hypothalamus 24 hours after uptake. This was accompanied by a statistically significant increase in malondialdehyde levels in most of the structures studied and at ACR doses.The highest increase in MDA was observed after 48 hours (45.12) and 192 hours (46.43) of exposure to ACR (40 mg/kg).	The assessment of Acetylcholinesterase activity in the muscles and hypothalamus of mice was very important due to the fact that many scientific reports indicate a direct effect of Acrylamide on peripheral nerves. It causes both their structural damage and negative physiological changes. After intraperitoneal injection of acrylamide, oxidative stress occurs in the body.
Prasad et al. [45]	Assessment of the potential of acrylamide to induce oxidative stress and neurotoxic effects in *Drosophila melanogaster* flies.	Adult male *Drosophilia melanogaster* flies have been exposed to dietary acrylamide.	ACR exposure resulted in dose-related and time-related mortality. The higher dose and the longer of the exposure to acrylamide, the worse results. Exposure to ACR caused locomotor deficits, severe oxidative stress, mitochondrial function, increased activity of acetylcholinesterase and decreased dopamine levels.	ACR-induced neurotoxicity may be mediated through an oxidative stress mechanism. ACR causes numerous physiological disorders, depending on the dose and time, such as abnormal modulation of nerve transmission by affecting the activity of AChE.
Couraud et al. [42]	To investigate the axonal transport of proteins in the proximal axons of the sciatic nerve in chickens as a result of acrylamide poisoning.	AChE occurs in nervous tissues of healthy chickens in four molecular forms (G1, G2, G4, A12). Three-day-old chickens were exposed to acrylamide at a dose of 100 mg/kg for 12 days. In the sciatic nerve of chickens poisoned with acrylamide, the action of individual forms of AChE was investigated.	Axonal transport of A12 was reduced by 60% and G4 by 21% due to ACR treatment.	ACR poisoning causes pathology in the action of some molecular forms of AChE. These forms can be considered sensitive markers of axonal transport and innervation phases.
Bai et al. [43]	To study the effect of exposure of the gastrocnemius motor plate in rats to ACR.	The rats were divided into groups. Different doses of ACR were administered to each group for 21 days. All rats were randomized into control groups, 9, 18 and 36 mg/kg.	As the dose of ACR exposure increased, more severe changes in the structure of muscle fibers and nerve endings were observed. Changes were observed in the structure of muscle fibers and nerve endings and a decrease in AChE content in motor plates.	ACR is toxic to the motor plate by altering the AChE content.
Ngo et al. [44]	To evaluate the effect of acrylamide on acetylcholinesterase.	The research was carried out on an electric eel. The study concerned the trapping of acetylcholinesterase from electric eel in a polyacrylamide gel.	AChE immobilized in the polyacrylamide gel showed a significantly reduced activity.	Acrylamide inhibits the activity of acetylcholinesterase.
